# Acupuncture for Primary Sjögren Syndrome (pSS) on symptomatic improvements: study protocol for a randomized controlled trial

**DOI:** 10.1186/s12906-017-1559-9

**Published:** 2017-01-19

**Authors:** Quan Jiang, Huadong Zhang, Ran Pang, Jinzhou Chen, Zhishun Liu, Xinyao Zhou

**Affiliations:** 1 Rheumatology Department, Guang’anmen Hospital, China Academy of Chinese Medical Sciences, Beijing, China; 2 Urology Department, Guang’anmen Hospital, China Academy of Chinese Medical Sciences, Beijing, China; 3 College of Acupuncture & Moxibustion, Beijing University of Chinese Medicine (BUCM), Beijing, China; 4 Acupuncture Department, Guang’anmen Hospital, China Academy of Chinese Medical Sciences, Beijing, China

**Keywords:** Sjogren’s syndrome, Acupuncture, Randomized controlled trial, Xerostomia, Fatigue, Pain

## Abstract

**Background:**

Currently, feasible medical treatments are hitherto not satisfying to relieve pSS symptoms, which concerns numbers of clinical doctors. Acupuncture seems to be an alternative to treat pSS and conduces to good symptomatic results. However further research is necessary. This trial is to investigate the efficacy of acupuncture on improving the key symptoms of pSS, which are dryness, pain and fatigue (DPF).

**Methods & Design:**

The study is designed as a randomized controlled trial of two arms with a single centre. We compare acupuncture with sham acupuncture on symptomatic improvements of pSS. A total of 120 pSS patients, aged at least 18, with DPF, will be randomly assigned to acupuncture or sham acupuncture groups, where they will have needle intervention for 8 weeks with 16 weeks of follow-up. Subjects will be assessed each time before interventions during the 8-week intervention, in week 8 after all interventions and in week 12, 16, 20 and 24 for follow-up with different measurements. The primary outcome are the proportions of subjects that have 30% or greater reduction in at least 2 out of 3 items of DPF in Numeric Analog Scale (NAS) scores (0 = the best, 10 = the severest), calculated between the baseline and the average scores of week 2 to 8. The secondary outcome are related to individual items of NAS scores, EULAR Sjögren’s Syndrome Patient Reported Index (ESSPRI), EULAR Sjögren’s Syndrome Disease Activity Index (ESSDAI), Schirmer test score and unstimulated salivary flow, serum Immunoglobulin G, A and M levels, Medical Outcome Study Short Form 36 Short-Form Health Survey (SF-36), Salivary glands ultrasounds, Hospital Anxiety and Depression (HAD) scale score. The secondary outcome scores are to be collected at baseline, in week 8, 16, and 24. Besides, individual items of NAS will also be collected in week 12 and 20. Moreover, subjects’ satisfaction and the proportion of the subjects who identified their allocation will also be measured and analyzed.

**Discussion:**

This study will be the first randomized and controlled pilot trial of acupuncture on alleviating the symptoms of pSS with relatively long-term follow-up. The result of the study might offer a new option to treat pSS and might be a clinical proof that acupuncture has beneficial effects on pSS.

**Trial registration:**

ClinicalTrials.gov: NCT02691377 (February 20, 2016)

## Background

Primary Sjögren’s syndrome (pSS) is a systematic autoimmune disease characterized by high lymphocytic infiltration in the exocrine glands such as salivary and lacrimal gland. The incidence in different populations varies from 0.5 to 2% [1]. There is a variety of clinical manifestations in pSS [2], in which, dryness, pain and fatigue (DPF) are considered as the 3 most urgent symptoms, and they affect patients quite much [3].

Current available medical treatments for pSS may lead to either serious adverse side effects, or failure to relieve symptoms. Thus, Bio-agents (e.g. rituximab, abatacept, belimumab), immunosuppression (e.g. and azathioprine, cyclophosphamide, mycophenolate mofetil) or corticosteroids should be cautiously applied clinically because of their safety and cost-effectiveness, although life-threatening complications after taking them are rarely seen. Even the most frequently prescribed medicine for pSS [4, 5], hydroxychloroquine, does not alleviate symptoms compared to the sham in a recent research [6]. Thus, some patients resort to complementary and alternative medicine, for example, acupuncture.

Acupuncture, as a non-pharmacologic therapy from Traditional Chinese Medicine, has been applied in a variety of disorders, including clinical symptoms of dryness [7]. Researches done in the past have shown the researchers’ recognition and expectation on acupuncture for dryness, and a research blank in “Acupuncture + Sjögren’s syndrome” as well [8, 9].

It seems that pSS patients have a good clinical response to acupuncture therapy in daily clinical practice, with acupoints and manipulations inherited from Professor Lu Zhizheng [10], one of the National Masters of Traditional Chinese Medicine (no more than 10 people in China), who has contributed himself to treating and studying rheumatoid diseases by using herbs and acupuncture for over 50 years. Our previous research indicates that acupuncture performs in a synergy with a particular formula of herbs, with which patients relieve from dryness of mouths and eyes, pain and fatigue, and it improves the quality of lives of pSS patients (it’s a result from our recent study not long after finished, which has been accepted by a journal but hasn’t been published yet). However, there are still questions not settled yet, such as how indeed is the effect of acupuncture on relieving symptoms of pSS statistically; if acupuncture works, what kind of patients will be benefit from it; and if not, why do some of the patients think they have symptomatic improvement after acupuncture. This study is designed to answer these questions.

## Methods & Design

### Study design

The study is a single-centre, double blinding and 24-week randomized and controlled trial with two arms, in one of which, needles will be inserted into previously set acupoints while sham acupuncture for the other group. 120 patients with symptomatic pSS are randomly assigned to acupuncture or sham acupuncture groups in 1:1 ratio. Needles are administered 3 times a week for the first 4 weeks, twice a week for the second 4 weeks, with 16-week of follow-up. All the allocation, intervention, randomization and analysis, etc., will be conducted in Guang’anmen (GAM) Hospital. The primary outcome is the two proportions of subjects in the two groups that have 30% or greater reductions in NAS scores in at least 2 out of the 3 DPF items [6]. They are calculated between baseline and the average scores between week 2 and week 8. The secondary outcome are related to individual items of NAS score at baseline, 3 times a week in week 0 to 8, and once a week at week 12, 16, 20, and 24, with EULAR Sjögren’s Syndrome Patient Reported Index (ESSPRI), EULAR Sjögren’s Syndrome Disease Activity Index (ESSDAI), Schirmer Test Score and Unstimulated Salivary Flow, Medical Outcome Study Short Form 36 Short-Form Health Survey (SF-36), Salivary glands ultrasounds, Hospital Anxiety and Depression (HAD) scale score, at baseline, week 8, 16 and 24. During the 8-week intervention, data of the last 24 h is collected each time before the intervention. Since there are only twice a week in week 4 to 8 for intervention but 3 times for NAS collection, the extra one is collected by phone.

### Randomization and blinding

Subjects eligible for participation will be randomly assigned to one of the following study groups, using the randomization software SAS (Statistical Analysis System) 9.2.

- penetrating acupuncture: 60 subjects
- sham acupuncture: 60 subjects

The statisticians will randomize assignments using a sequence of number randomly generated by the SAS (Statistical Analysis System) 9.2, done by a third organization, a Statistical Department of State Standardized Traditional Chinese Medicine Pharmacology Clinic (SDSSTCMPC) granted by State Food and Drug Administration (SFDA). The sequence will be recorded and can be displayed when necessary. These assignments will be sent to a study member, who will put them into sealed, opaque envelopes with dates and signature labels over the seals. And this study member will not take part in any of patient interviews, acupuncture intervention, data collection, or statistical analysis.

Before eligible subjects are randomized into different groups, informed consent contract will be obtained from each participant and they will be interviewed to ensure their baseline data recorded. Then they will allocate and come to see the acupuncturist who will open the consecutive randomization envelope, and then apply acupuncture or sham one according to the assignment without informing the subjects of their assignments. As the only one knowing the group assignments of subjects, the acupuncturist will not participate in any of the interviewing, data collection, or statistical analysis. Every time before the intervention, except the first time, which is baseline, the subjects will come to the interviewing room where the case report form (CRF) with NAS scores will be filled with their feedback on NAS scores about last 24 h instead of a time point. After the intervention, the schedule for their acupuncture and interviewing for next time (including date and time) will be informed.

### Patients

#### Inclusion criteria

- Age 18+
- To meet the American-European Consensus Group Criteria for primary Sjögren syndrome [11]
- Symptoms have been appearing for less than 5 years; signed a written informed consent form
- Without taking pSS medicines, or have not been taking pSS medicines for at least 4 weeks before the recruitment, or have been taking the same kinds and doses of pSS medicines (non-steroidalanti-inflammatory drugs, NSAIDs; oral taken cortical hormone; pilocarpine; artificial eye liquid; herbal decoctions) for more than 4 weeks before the recruitment, or have been taking the same kinds and doses of other pSS medicines (tripterygium wilfordii; methotrexate; cyclophosphamide; cyclosporin; azathioprine; hydroxychloroquine sulfate) for more than 6 months before the recruitment.

#### Exclusion criteria

- Secondary Sjogren syndrome
- Have been having serious systemic disorders recently (lymphoma; central nervous system, renal, or pulmonary involvement; myositis; or vasculitis) or severe renal or liver failure
- Have had acupuncture treatments in last 20 days
- With acupuncture contraindications (allergy to metals, skin lesions on relative acupoints, etc.)
- Subjects who participate in any other clinical trials in last 30 days before recruitment
- Currently or planning to be pregnant, or at risk of being pregnant without contraception
- Subjects with a physical or psychological problem, which may confound the trial results, interfere with other subject’s participation, or may make it risky to follow the investigators’ requirements
- Subjects known for or persistent in drug or alcohol abuses

### Recruitment strategies

Patients will be recruited through a number of advertising strategies, with the information of physicians and the details and requirements of the trial. Posters in GAM Hospital, and advertisements on the online official medical medias, such as websites, Weibo (similar to twitter in China), and Wechat (similar to Facebook in China) of Rheumatology Department of GAM Hospital, will also be applied for recruitment. Also, another main recruiting way is that patients comes from outpatient service.

### Intervention

Since sham control is a useful method for determining the effectiveness of a therapy, we will choose a sham needle [12] that has a blunt tip and achieves no skin penetration. To apply the sham needles produces a good subject blinding effect, the reason of which is that they have a similar appearance to conventional acupuncture needles, however, without skin penetration. Also, we put plastic pads on the acupoints before to cover the areas so as to keep the patients blinded to if or not there’s penetration.

### Description of sham acupuncture

**Fig. 1:**
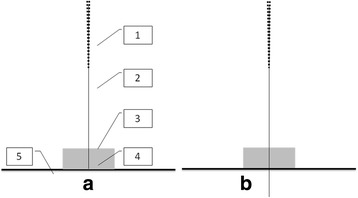
The sham and real acupuncture we use in this study. **a**: sham acupuncture as control; b: real acupuncture. 1. needle handle; 2. needle body; 3. adhesive pad; 4. blunt tip; 5. Cutis

The sham needle is 0.30mm wide and 25mm long. It consists of an adhesive pad with a blunt tip (Fig. 1). The adhesive pad is made of a sterile cylindrical polyethylene foam (diameter 10mm and height 5mm) with a double-sided adhesive tape at the bottom. The adhesive pad has 2 functions: one is holding the sham needle on the acupoints when used in sham acupuncture and the other function is assisting the implementation of blinding when used in both sham and real acupuncture. In all, the only difference between normal and sham needles is that the sham ones tips are not tapering.

### Process of the intervention

**Fig. 2:**
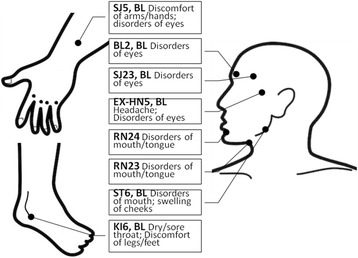
The acupoints we picked in this study and their indications

All of the subjects will be told that we are going to evaluate the effects of “a new kind of acupuncture” researching on “deep penetrating needles and shallow penetrating needles” on pSS, without mentioning sham, pseudo or the penetration status. The experienced acupuncturist will be trained on the sham needles and will apply both the sham and real needles throughout the whole trial. Each time before the intervention, subjects will be asked to meet the interviewer in another room to provide their answers on CRF with the feelings of the last 24 h before the intervention, excluding the first intervention, whose data will be the baseline. Acupoints and manipulations are the same in 2 groups (Fig. 2), with bilateral Waiguan (SJ5) with reducing method; bilateral Zhaohai (KI6) with reinforcing method; Chengjiang (RN24), Lianquan (RN23), bilateral Taiyang (EX-HN5), bilateral Cuanzhu (BL2), bilateral Sizhukong (SJ23), and bilateral Jiache(ST6) with even method. During the manipulations, twirling, lifting, and thrusting to elicit Deqi or needle sensation (a composite of unique sensations interpreted as the flow of qi induced by acupuncture and essential for clinical efficacy [13]), will be applied, and the needles will be remained for 20 min. Subjects will receive acupuncture or sham acupuncture 3 times a week for the first 4 weeks, twice a week for the second 4 weeks, followed by 16 weeks of observation without treatments. For week 4 to 8, intervention will only be conducted twice a week, therefore one more interview for NAS scores every week will be on the phone.

#### Primary outcome

The numeric analog scale (NAS) [14] scores of DPF (0 = the best, 10 = the severest) will be measured at the baseline, three times a week in week 1 to 8, and once a week in week 12, 16, 20, and 24. By the comparison of the average scores of week 2 to 8 with those of the baseline of the two arms, the proportions of the subjects in two groups that have 30% or greater reductions in NAS scores in at least 2 out of 3 DPF items, will be the primary outcome.

Subjects will be asked to provide every NAS score based on the overall situation of the past 24 h [15] rather than a point of time. And we will collect NAS scores 3 times a week in week 1 to 8 (24 times in total).

**Fig. 3:**
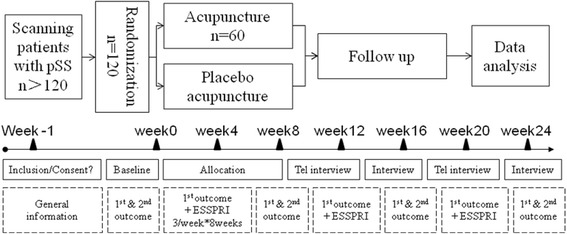
Trial flow & study design. General information = age, sex, weight, time when symptoms emerge, time when diagnosed, anti-SSA antibodies, abnormal Schirmer test results, decreased unstimulated salivary flow, previous systemic involvement, previous treatment with another immunosuppressant, current systemic involvement. 1st outcome = numeric analog scale (NAS) (Dryness, Pain and Fatigue). 2nd outcome = EULAR Sjogren Syndrome Patient Reported Index (ESSPRI), EULAR Sjogren Syndrome Disease Activity Index (ESSDAI), Medical Outcome Study Short From 36 Short-Form Health Survey (SF-36), Hospital Anxiety and Depression (HAD), Serum Immunoglobulin (IgG), IgA and IgM, Schirner test score and unstimulated salivary flow, salivary glands untrasounds

The mentioned outcome measurements will be assessed at the marked time points respectively (Fig. 3).

#### Secondary outcome

Secondary outcomes include each items of NAS scores from week 0 to 8. These items will be measured individually at baseline, 3 times a week in week 1 to 8, once a week in week 12, 16, 20, 24. The secondary outcomes also include:

- EULAR Sjögren’s Syndrome Patient Reported Index (ESSPRI) [16] is a very simple index completed by patients to measure symptoms in pSS. With good construction validity, it is used as an outcome measure in clinical trials. There is a 0–10 numerical scale for each of the four domains, dryness, discomfort (including pain), global fatigue and mental fatigue, which are gathered to form the ESSPRI. For sicca features, six components were identified, which are ocular, oral, skin, nasal, tracheal and vaginal.
- EULAR Sjögren’s Syndrome Disease Activity Index (ESSDAI) [17, 18] is a clinical index completed by physicians to measure disease activity in patients with pSS. There are 12 organ-specific ‘domains’ contributing to disease activity. For each domain, features of disease activity are classified into three or four levels according to their severity.
- Schirmer test score and unstimulated salivary flow [19]: these two tests are the objective reflection to the lacrimal gland and salivary glands, the results of which can be greatly impacted by the operator. Thus, there will be a technician performing these two tests during the whole trial to maintain the homogeneity of the operation.
- Serum Immunoglobulin G, A, M levels [20]: Immunoglobulin G (IgG) is commonly believed in clinic a reflection of pSS symptom activities and a study shows weak associations between increasing fatigue and serum IgG, and the pro-inflammatory cytokine interleukin-17 (IL-17), were observed.
- Medical Outcome Study Short Form 36 Short-Form Health Survey (SF-36) [21, 22] is a self-administered questionnaire with 36 items, which assesses the concepts of physical function, role limitations due to physical problems, social function, body pain, general mental health, role limitations due to emotional problems, vitality, and general health perceptions. Note that both physical and mental component summaries can be combined. Scores range from 0 to 100, with higher scores indicating better health status [23].
- Salivary glands ultrasounds [24]: image of salivary glands structures. This can be quantified by a form based on different features.
- Hospital Anxiety and Depression (HAD) scale score [25]: A self-assessment scale which has been developed and found as a reliable instrument for detecting states of depression and anxiety in the setting of an hospital medical outpatient clinic.

For the secondary outcomes listed above, the measurements are conducted at baseline, in week 8, 16 and 24. Besides, the satisfaction of patients in two groups and the proportion of the subjects who identified their allocation will be measured in week 8.

Additionally, we will assess the patients’ satisfaction in week 24 by two questions. 1) “Would you like to come for more treatments or recommend it to your friends/relatives if they have similar symptoms?” The answer will be either “yes” or “no”. 2) "What is your feeling for the series of treatment?” The answers will include “excellent”, “good”, “satisfactory”, “It’s okay” and “poor”. The difference in satisfaction between two groups will be analyzed.

To evaluate the success rate of blinding, we will ask subjects in week 8 “how deep do you think the needles went into your skin?” The answer will be “deep”, “shallow” or “no penetration”. The subjects in the two groups whose answers are either “deep” or “shallow” will be considered to be blinded successfully, while “no penetration” unsuccessfully, and the proportions of them are going to be compared.

### Sample size

Sample size will be decided by the primary outcome. According to our unpublished pilot study and literature review [6], the proportions of patients with a more than 30% improvement in NAS scores in at least 2 out of 3 DPF items for acupuncture and placebo is 45% and 17.9% respectively. We use the proportion for placebo instead of the one in sham acupuncture group, because no data are available for sham acupuncture so far. A sample size of 52 for each group will be sufficient to detect a 2% null difference in proportion, with a two-sided 5% level of significance, and a power of 80%. The number of subjects is increased to 60 in each group (total of 120) when a drop-out rate of 15% is taken into consideration.

### Statistical analysis

For data analysis, statisticians and investigators will be blinded for the patients’ allocation. Statistical analysis will be performed using SPSS Ver.20 (SPSS Inc., Chicago, IL, USA) software. Enumeration data will be described as frequency and composition ratio. Continuous data will be presented by Mean ± Standard Deviation (M ± SD) if they meet normal distribution, otherwise they will be expressed as Medians ± Interquartile Range (M ± IQR). Categorical data will be presented by frequency and percentage. The analyses of efficacy will be based on the intention-to-treat (ITT) analysis set, which was defined as patients who receive at least one treatment. Missing data for the primary outcome will be treated by multiple imputation under chained equation, with m = 50 imputations. The covariates used to generate the multiple imputed data sets are age, sex, NAS scores of DPF, ESSDAI, ESSPRI, salivary flow rate, and Schirmer test score. For comparison with the baseline, a repeated measures data analysis of variance (ANOVA) or Wilcoxon signed rank test will be used for continuous data, whereas Chi-square test or Fisher exact test will be used for categorical data. In terms of comparison between the two groups, Chi-square test or Fisher exact test will be used for primary outcomes. For secondary outcomes, the analysis of covariance (ANCOVA) will be used if the residual are normal distributed, otherwise Wilcoxon rank sum test will be applied. To access the success of blinding, the proportion of subjects who are successfully blinded will be compared by Chi-square test or Fisher exact test. And nonparametric test will be used to analyze satisfaction. *P* <0.05 with two-tailed test indicates statistical significance.

## Discussion

This trial is designed to assess the effects of acupuncture on DPF of pSS, with the alleviation of the symptoms as a clinical priority when severe complications absent in most cases. In this study, we will evaluate if the effect of acupuncture is not worse than that of sham; the characteristics of the patients who have better responses to acupuncture at the reasonable end points in multiple perspectives, covering the subjectivity and objectivity of patients’ feedback. The objective feedbacks are the structure and function of salivary glands, the disease activity, and complications of pSS. The subjective feedbacks include feelings of DPF and emotions.

### Pragmatic inclusion criteria

Inclusion criteria were held pragmatically to facilitate screening and recruitment. The symptom duration is set to be “less than 5 years” in the inclusion criteria, because the structure and function of the glands would be aggravated with the course of disease, which would be more difficult to be reversed [25] if it’s over 5 years. To make this trial more similar to the real world where it’s fairly common for hospitals to accept patients while they are talking other medicine, we therefore recruit those taking medicines. However on the other hand, to maintain the interference from taken medicines during this study as low as possible, we have a very strict recruitment criteria listed earlier in this article in case the medicines influence the results of the study.

### Sham needle as a control procedure

With a blunt end and achieving no skin penetration, the sham needle that we choose as a control procedure in this trial has the same appearance and gives a likely sense of penetration to a real one (Fig. 1). In a crossover study [12], needle penetration was felt by 88.3–100% of volunteers receiving this kind of sham acupuncture which are not penetrating in fact, while by 95–98.3% of volunteers receiving real acupuncture penetrating. The results make it probable to keep the subjects unaware of penetration status. To make the end of the needles invisible to subjects when conducting intervention, which makes subjects to feel the seeming penetration without knowing if the needles really penetrates through the skin or not, we will place sticky plastic pads on the acupoints and then we perform real/sham needles through the pads and then either penetrating through skin or not depends on which assignment the subjects are in. In result, subjects will be blinded from their assignments and would have the same psychological expectations from the interventions.

### Informing strategy

Since many of the subjects in China may have basic ideas of acupuncture, they would notice the difference between the needles used in this trial with plastic pads and the traditional ones applied clinically without (Fig. 2). Thus, we will inform subjects of the words “a new method of acupuncture”. “New” refers to the pads we put on acupoints before we stick needles and that’s for explaining to subjects in case the differences concern them. With obedience to the ethics principle, we will also mention that we are going to assign them into deep penetrating group and shallow penetrating group based on the “new method of acupuncture”. This expression can avoid telling pseudo or sham. If not doing so it may induce a higher psychological expectation in patients of study group, which can be beneficial to the study group’s result. To keep this particular bias out, we inform subjects of “a new method of acupuncture”, meanwhile, obeying ethic requirements.

### Efforts to minimize some other bias

Besides the details of sham needles and the inform strategy, there are a lot of other efforts we take to minimize the possible bias. We will give subjects acupuncture in one room and interview them in another, so that the physicians, the interviewer, the data collectors, and the statistics will be blind to the group allocations. What else, to ensure the consistency of manipulation in repeated courses of real/sham acupuncture, we will train the acupuncturist and write a standard operation procedure (SOP) and this acupuncturist will stick to SOP throughout the whole study. It is designed to be based on a specialists’ consensus, which is 3 times of acupuncture a week in the first 4 weeks followed by twice a week in the second 4 weeks. It obeys the acting rules of this traditional therapy. And the subjects will be followed up by another 16 weeks besides 8 weeks of acupuncture, in order to ensure the relatively long-term effects to be observed rather than only the short-term ones.

When NAS scores are collected in CRF, a general pSS condition of the last 24 h will be asked to avoid temporary diverse interferences only existing during the interviews, such as mental relaxation in weekend, weather changing, and unexpected events in life, etc. Schirmer test, unstimulated salivary flow, and salivary grand ultrasound will be performed by the same technicians to maintain the consistency, before and after the 8-week intervention and during the follow-up, which also reduces bias.

### The selected acupoints

We selected acupoints based on the experiences of Professor Lu Zhizheng. The given acupoints will be a practical option for both domestic and international doctors and researchers for either their clinical prescriptions or studies. According to TCM theory, Taiyang (EX-HN5), Cuanzhu (BL2), and Sizhukong (SJ23) are functioning in the eye disorders. Chengjiang (RN24), Lianquan (RN23) and Jiache (ST6) take actions on dry mouth. Waiguan (SJ5) and Zhaohai (KI6), as the points in the corresponding meridians distal to the local areas, act not only on dryness of eyes and month, but also on pain and fatigue. Thus, with these acupoints mentioned above, this study is designed to evaluate the effect of acupuncture on symptoms of pSS patients since the acupoints work well in coordination theoretically and seemingly clinically.

### Limitations

Limitations include the restricted sample size and single centre. Although the study takes place in GAM hospital in Beijing, it is not possible to limit subjects into only local citizens as there is a large number of people immigrating to Beijing from all over China and so do the patients. Thus, the differences of people in different areas of China may interfere the outcomes of the trial. Although we only accept subjects having less than 5-year of symptomatic pSS, different durations of having it may also interfere the outcomes. Besides, we can’t eliminate the medicine interferences from those who are taking other medicines during the study. We don’t know what the informing strategy would actually and exactly bring to subjects mentally by telling them we are researching on a “new” method of acupuncture, although our initial purpose of doing so is to make subjects to have the same expectations from their interventions. Another limitation is that the acupuncturist knows assignments and he conducts, in which case the communication between the acupuncturist and subjects may, more or less, change the expectations of subjects, which would cause bias. What’s more, the acupoints studied in this trial are constant. It is not the way of applying acupuncture clinically in TCM way, in which case we wouldn’t know how acupuncture would act if it’s done with Differentiation Method, a way of diagnosis in TCM, which also directs treatments. Finally, if there is a third group by standard care, the result would be more convincing; while, standard treatments based on the guideline of pSS had not been established yet before the start of this study, and our fund is not financially supportive to set a third group of cohort.

## Abbreviations

pSS: Primary Sjögren syndrome
DPF: Dryness, pain and fatigue
NAS: Numeric analog scale
CPF: Case report form
SOP: Standard operation procedure
TCM: Traditional Chinese medicine
GAM: Guang’anmen
BUCM: Beijing University of Chinese Medicine
SDSSTCMPC: Statistical Department of State Standardized Traditional Chinese Medicine Pharmacology Laboratory
SFDA: State of food and drug administration
IgG: Immunoglobulin G
SAS: Statistical analysis system
ITT: Intend to treat
